# Total antioxidant status levels in malaria: a systematic review and meta-analysis

**DOI:** 10.1186/s12936-024-05003-z

**Published:** 2024-06-26

**Authors:** Kwuntida Uthaisar Kotepui, Aongart Mahittikorn, Wanida Mala, Supakanya Lasom, Frederick Ramirez Masangkay, Hideyuki J. Majima, Manas Kotepui

**Affiliations:** 1 Medical Technology, Faculty of Science, Nakhon Phanom University, Nakhon Phanom, 48000 Thailand; 2 Department of Protozoology, Faculty of Tropical Medicine, Mahidol University, Bangkok, Thailand; 3 Department of Medical Technology, School of Allied Health Sciences, University of Phayao, Phayao, Thailand; 4 Department of Medical Technology, Faculty of Pharmacy, University of Santo Tomas, Manila, Philippines; 5 Medical Technology, School of Allied Health Sciences, Walailak University, Tha Sala, Nakhon Si Thammarat, Thailand

**Keywords:** Malaria, *Plasmodium*, Oxidative stress, Antioxidants, Total antioxidant status, Total antioxidant capacity, Total antioxidant defense

## Abstract

**Background:**

Malaria, a severe health threat, significantly affects total antioxidant status (TAS) levels, leading to considerable oxidative stress. This systematic review and meta-analysis aimed to delineate differences in TAS levels between malaria patients and healthy controls, and assess correlations between disease severity and parasite density.

**Methods:**

The systematic review was registered with the International Prospective Register of Systematic Reviews (PROSPERO) under registration number CRD42023448761. A comprehensive literature search was conducted in databases such as Embase, MEDLINE, Journals@Ovid, PubMed, Scopus, ProQuest, and Google Scholar to identify studies reporting data on TAS levels in malaria patients. Data from the included studies were analysed both qualitatively and quantitatively. Differences in TAS levels between malaria patients and controls were pooled using a random effects model, with Hedges' g as the effect size measure.

**Results:**

Of 1796 identified records, 20 studies met the inclusion criteria. The qualitative synthesis of these studies revealed a marked decrease in TAS levels in patients with malaria compared to non-malaria cases. The meta-analysis results showed a significant decrease in TAS levels in patients with malaria compared to non-malaria cases (*P* < 0.01, Hedges’ g: − 2.75, 95% CI − 3.72 to −1.78, *I*^*2*^: 98.16%, 13 studies), suggesting elevated oxidative stress in these patients. Subgroup analyses revealed that TAS level variations were significantly influenced by geographical region, age group, *Plasmodium* species, and method for measuring TAS. Notably, TAS levels were significantly lower in severe malaria cases and those with high parasite density, indicating a potential relationship between oxidative stress and disease severity.

**Conclusion:**

This study highlights the potential utility of TAS as a biomarker for disease risk and severity in malaria. The significant decrease in TAS levels in malaria patients compared to controls implies increased oxidative stress. Further well-designed, large-scale studies are warranted to validate these findings and elucidate the intricate mechanisms linking TAS and malaria.

**Supplementary Information:**

The online version contains supplementary material available at 10.1186/s12936-024-05003-z.

## Background

Malaria, a life-threatening disease caused by *Plasmodium* parasites and transmitted via the bites of female *Anopheles* mosquitoes, continues to pose a significant global health threat [1]. Five species of *Plasmodium*, namely *Plasmodium falciparum*, *Plasmodium vivax*, *Plasmodium ovale* spp., *Plasmodium malariae*, and *Plasmodium knowlesi*, are known to infect humans, each leading to different disease forms with varied severity and clinical manifestations [1]. Among these, *P. falciparum* is most commonly associated with severe malaria, causing the majority of malaria-related fatalities worldwide [1, 2]. The clinical manifestations of *Plasmodium* infection, which can range from mild symptoms to severe and often fatal complications, are significantly influenced by a combination of factors: the infecting *Plasmodium* species, the host's immune response, and the complex interaction between the parasite and the host [3–5].

A vital aspect of the host–pathogen interaction in malaria pertains to oxidative stress, a state arising from an imbalance between the production of reactive oxygen species (ROS) and the detoxification capacity of the antioxidant system [6, 7]. The production of ROS initiates with the swift intake of oxygen and the activation of NADPH oxidase (NOX), forming the superoxide anion (${\text{O}}_{2}^{ - \cdot }$). [8]. This anion is then rapidly transformed into hydrogen peroxide (H_2_O_2_) by the enzyme superoxide dismutase (SOD) [8]. During malaria infection, ROS are generated at elevated levels through multiple mechanisms. These include the host's immune response aimed at controlling the infection and the metabolic activity of the parasite itself, which produces ROS as byproducts [6, 9]. Elevated ROS levels can inflict extensive cellular damage, such as lipid peroxidation, protein modification, and DNA damage, thereby exacerbating the pathological conditions associated with malaria [6, 9].

Antioxidants, including enzymatic antioxidants like SOD, catalase (CAT), and glutathione peroxidase (GPx), as well as non-enzymatic antioxidants such as glutathione and vitamins C and E, play a crucial role in neutralizing ROS and repairing oxidative damage [10–13]. These antioxidants act by either scavenging the ROS directly or repairing oxidative damage, thereby maintaining cellular homeostasis. Assessing total antioxidant status (TAS) or total antioxidant capacity (TAC) incorporates the collective effect of all antioxidants present in plasma and body fluids, thus offering a comprehensive parameter that extends beyond the sum of individual measurable antioxidants [14].

The alterations in TAS during malaria infection are influenced by the increased oxidative stress and the body’s antioxidant defense response. In malaria, TAS levels may decrease due to the overwhelming ROS production that depletes antioxidant reserves or may show compensatory increases as the body attempts to counteract oxidative stress [15–17]. However, reports on TAS levels in malaria patients have shown inconsistencies, often based on studies with limited participant numbers and varied methodologies. Investigating the alterations in TAS levels and their role in the pathogenesis of malaria could be key to developing more effective management strategies. The present systematic review and meta-analysis were conducted to discern the difference in TAS levels between patients with malaria and non-malaria controls and to analyze the relationship between TAS, disease severity, and parasite density.

## Methods

### Protocol and registrations

The systematic review was registered with the International Prospective Register of Systematic Reviews (PROSPERO) under the registration number CRD42023448761, and the reports followed the Preferred Reporting Items for Systematic Reviews and Meta-Analyses (PRISMA) statement [18].

### Review question

The systematic review addressed the research question using the Population, Exposure, and Outcome (PEO) framework [19] to investigate the association between TAS and *Plasmodium* infections. Population refers to the participants enrolled in each study; Exposure was defined as malaria (any severity); and Outcome pertains to TAS levels.

### Search strategy

The systematic literature searches were conducted in primary databases, such as PubMed, Embase, Medline, Scopus, and Journals@Ovid, as well as ProQuest, to identify all relevant articles published up to July 25, 2023. The search strategy involved a combination of Medical Subject Heading (MeSH) terms and keywords related to TAS and malaria, as follows: “(“total antioxidant status” OR TAS OR “oxidative stress index” OR OSI OR “total antioxidant capacity” OR TAC OR “redox status” OR “total antioxidant” OR “total oxidant” OR “total oxidant status” OR TOS OR “total antioxidant defense” OR “total antioxidant power” OR “Ferric Reducing Ability of Plasma” OR FRAP OR “overall antioxidant status” OR “overall antioxidant capacity” OR “overall antioxidant power” OR “overall antioxidant defense”) AND (malaria OR *Plasmodium* OR “*Plasmodium* Infection” OR “Remittent Fever” OR “Marsh Fever” OR Paludism)” (Table S1). Searches were also performed in Google Scholar and the reference lists of selected studies to ensure no relevant studies were missed.

### Eligibility criteria

The inclusion criteria were: (i) studies published up to 2023; (ii) studies with human participants; (iii) studies reporting data on TAS levels in individuals with malaria; (iv) studies using case–control, cross-sectional, or cohort study designs; (v) studies conducted in any geographical region of the world; and (vi) studies utilizing various methods for malaria detection (e.g., microscopy, rapid diagnostic test, polymerase chain reaction). The exclusion criteria were: (i) studies not relevant to the research question (e.g., not reporting on TAS levels in individuals with malaria); (ii) studies based on in vitro or in vivo models rather than human participants; (iii) articles that were reviews, mosquito studies, or focused on assay performance rather than original research; (iv) non-original articles such as systematic reviews, case reports, or commentaries; and (v) studies with incomplete data or insufficient details for data synthesis.

### Study selection and data extraction

After removing duplicates, all identified studies were screened based on their titles and abstracts for potential relevance. Full texts of potentially relevant studies were obtained and further assessed for eligibility based on pre-specified inclusion and exclusion criteria. For each eligible study, pertinent information was extracted using a standardized data extraction form. This typically includes authors' names, publication year, study design, geographical areas, year of sample collection, sample size, patient characteristics (such as age, gender, and disease severity), methods for TAS measurement, TAS levels in both malaria and non-malaria cases, and any other relevant results. The study selection and data extraction were conducted independently by two authors. Any discrepancies were resolved through discussion or consultation with a third reviewer.

### Quality assessment

The quality of the included studies was assessed using the Joanna Briggs Institute (JBI) Critical Appraisal Checklist for case–control, cross-sectional, and cohort study designs, which evaluates the methodological quality of studies included in systematic reviews [20]. For case–control studies, the checklist assesses factors such as the clarity and appropriateness of the case definition, selection and matching of controls, measurement of exposure, identification of confounding factors, and validity of outcomes. Similarly, for cross-sectional studies, the checklist considers the representativeness of the sample, measurement of exposure, assessment of outcomes, identification of confounders, and appropriateness of statistical methods. For cohort studies, the checklist examines the recruitment of participants to minimize selection bias, measurement of exposure, assessment of outcomes, handling of confounders, adequacy of follow-up period, addressing of losses to follow-up, presentation of results, and appropriateness of statistical methods. Each item was rated as "Yes," "No," "Unclear," or "Not applicable" to indicate the presence or absence of specific methodological components. Two authors conducted the quality assessment independently, with any discrepancies resolved through discussion or consultation with a third author.

### Data syntheses

Data extracted from each study were synthesized both qualitatively and quantitatively. The qualitative synthesis was conducted by narratively summarizing the findings of individual studies reporting similar outcomes. The quantitative synthesis of the difference in TAS levels between malaria and non-malaria cases was pooled using the random effects model, with Hedges' g employed as an effect size estimate. The statistical heterogeneity among the studies was evaluated using the *I*^*2*^ statistic [21]. Random-effects meta-analysis models were employed if the *I*^*2*^ value exceeded 50%, presupposing variability in the actual effect among the studies. A meta-regression analysis was performed to evaluate publication year, study design, geographical location, age group, *Plasmodium* species, and method for measuring TAS as potential covariates influencing TAS levels [22]. The subgroup analyses were conducted to explore how these covariates influenced the pooled effect estimate. A leave-one-out meta-analysis was performed to assess the stability of the results with each iterative re-run of the analysis [23]. The potential for publication bias was assessed through visual inspection of funnel plots and statistical tests, including Egger's regression test [24, 25]. All statistical analyses were performed using Stata version 18.0 (StataCorp, TX, USA).

## Results

### Search results

**Fig. 1:**
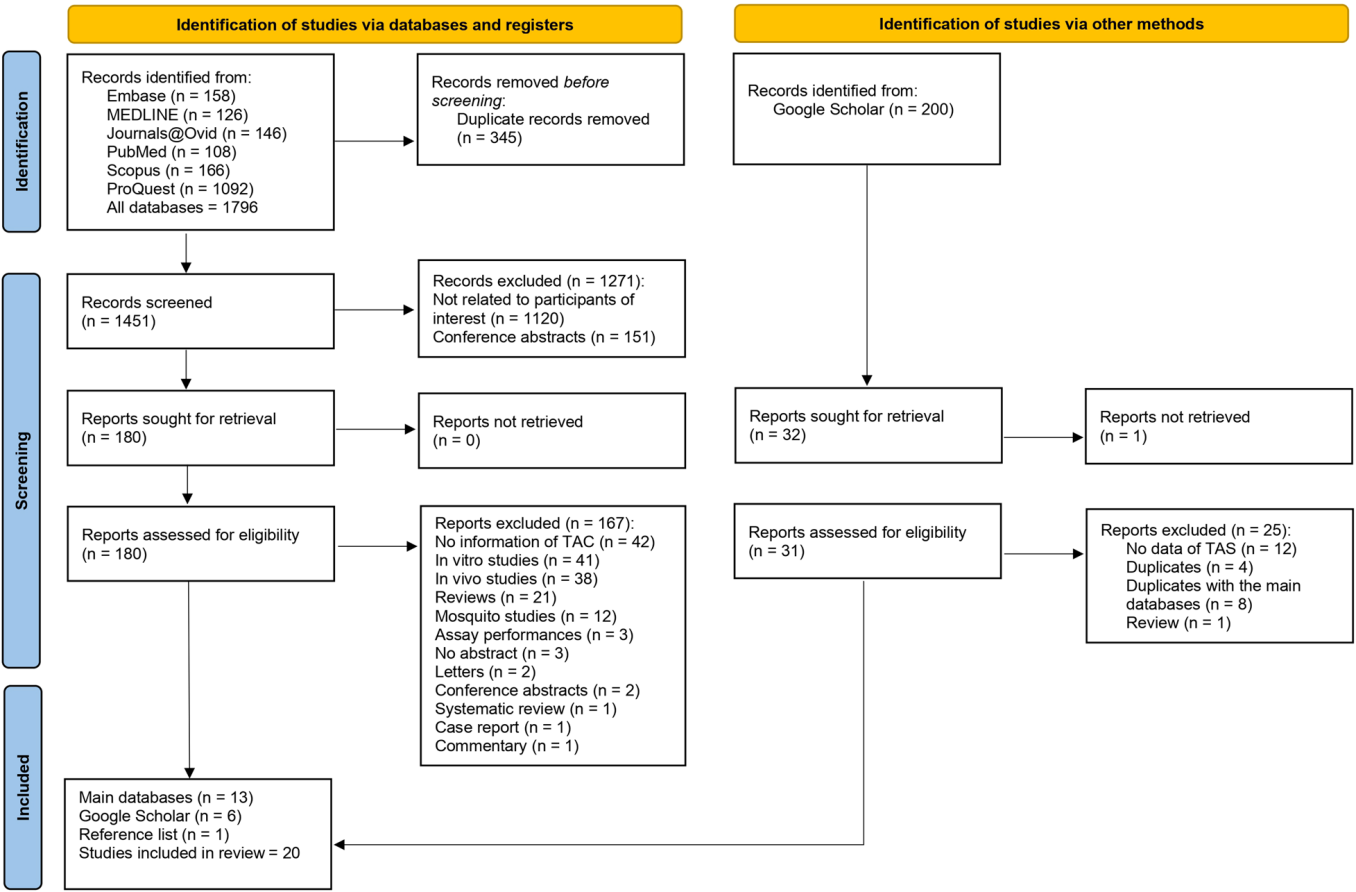
Study flow diagram

The initial search yielded a total of 1796 records. After the exclusion of 345 duplicate records, 1451 records remained and were then screened. During the screening phase, 1271 records were excluded for reasons such as not being related to the participants of interest (1120 records) or being conference abstracts (151 records). As a result, 180 reports were deemed relevant for retrieval. Out of these 180 reports, further scrutiny led to the exclusion of 167 for various reasons, such as lack of TAC data, being in vitro or in vivo studies, being reviews or mosquito studies, or presenting assay performances. Others were excluded because they lacked abstracts, were letters or conference abstracts, or were non-original articles such as systematic reviews, case reports, or commentaries. After these exclusions, a total of 13 studies were retrieved from the main databases [15–17, 26–35]. This list was supplemented with six studies from Google Scholar [36–41] and one additional study identified from a reference list [42], bringing the total to 20 studies [15–17, 26–42] (Fig. 1).

### Characteristics of the included studies

Table 1 delineates the characteristics of the 20 studies scrutinized for analysis. Predominantly published between 2010 and 2023 (75% combined), these studies primarily employed case–control (55%) and cross-sectional (40%) designs. Geographically, a significant portion of the studies (70%) were conducted in Africa, with Nigeria being the focal point for half of these studies. Research in Asia and South America contributed 20% and 10%, respectively. Regarding the *Plasmodium* species examined, *P. falciparum* was the most commonly studied species (55%). The majority of studies (60%) targeted adults, while the remaining studies targeted children (10%), all age groups (10%), cord blood (5%), and did not specify age groups (15%). In the context of clinical malaria, the symptomatic category was the most examined (45%), with a considerable number of studies (45%) not specifying the clinical status. The microscopic method was the most commonly used for malaria detection (55%), with some studies using combinations of microscopy with rapid diagnostic tests (RDT) or polymerase chain reaction (PCR). However, 15% of the studies did not disclose their detection methods. Ferric reducing antioxidant power (FRAP) was the most commonly used method for measuring TAS levels (30%), followed by Thiobarbituric acid reactive substances (TBARS) (20%) and Trolox equivalent antioxidant capacity (TAC) (15%). Details of individual studies are shown in Table S2.

**Table 1:** Characteristics of studies included in the review

Characteristics	No. (20 studies)	**%**
**Publication year**		
2020–2023	7	35
2010–2019	8	40
2000–2009	5	25
**Study designs**		
Case–control studies	11	55
Cross-sectional studies	8	40
Cohort study	1	5
**Study areas**		
**Africa**	**14**	**70**
Nigeria	10	50
Cameroon	2	10
Ethiopia	1	5
Ghana	1	5
**Asia**	**4**	**20**
India	4	20
**South America**	**2**	**10**
Brazil	1	5
Colombia	1	5
*Plasmodium* **species**		
* P. falciparum*	11	55
* P. falciparum, P. vivax*	3	15
* P. vivax*	2	10
* P. falciparum, P. vivax,* mixed infections	1	5
Not specified	3	15
**Age group**		
Adults	12	60
Children	2	10
All age groups	2	10
Cord blood at delivery	1	5
Not specified	3	15
**Clinical malaria**		
Symptomatic	9	45
Asymptomatic	1	5
Symptomatic, asymptomatic	1	5
Not specified	9	45
**Methods for malaria detection**		
Microscopy	11	55
Microscopy/RDT	4	20
Microscopy/PCR	1	5
RDT	1	5
Not specified	3	15
**Methods for measuring TAS levels**		
Ferric reducing antioxidant power	6	30
Thiobarbituric acid reactive substances	4	20
Trolox equivalent antioxidant capacity	3	15
2,2-azinobis 3-ethylbenzothiazoline-6-sulfonate	3	15
Commercial kits	2	10
*N*,*N*-dimethyl-*p*-phenylenediamine sulphate	1	5
Not specified	1	5

*RDT* rapid diagnostic test, *PCR* polymerase chain reaction

Bold texts indicate the main characteristics of included studies

### Quality of studies

All 11 case–control studies met most quality assessment criteria, including comparable groups, appropriate matching, consistent criteria for case identification, reliable exposure measurement, and valid outcome assessment [15, 26–29, 32, 33, 37, 38, 40, 41] (Table S3). Some studies addressed confounding factors and provided strategies to deal with them [27–29, 40, 41], while others did not. A few studies had exposure periods long enough to be meaningful [15, 37, 38], but not all. Eight cross-sectional studies generally met the following criteria [16, 17, 30, 31, 34–36, 39], but one study lacked a detailed setting description [39], and some studies did not report on confounding factors or provide explicit strategies to address them [17, 30, 31, 35, 36, 39]. A cohort study lacked strategies to deal with confounding factors and to address incomplete follow-up [42]. The appropriate statistical analysis methods were unclear in all studies.

### Synthesis of total antioxidant status levels in malaria patients

The findings across several studies denote a marked decrease in TAS levels in patients with malaria compared to non-malaria cases. This trend was observed in several studies [15, 17, 26, 27, 29, 32, 33, 35, 37–42]. Furthermore, some studies also identified a negative association between TAS levels and parasite density [17, 33, 40]. Moreover, Aqeel et al*.* found that TAS levels significantly dropped in severe malaria cases compared to non-severe ones [28]. Aqeel et al*.* further differentiated between *Plasmodium* species, showing a significant decrease in TAS levels in *P. vivax* malaria patients compared to *P. falciparum* malaria patients and non-malaria cases, but no significant difference between *P. falciparum* patients and non-malaria cases [27]. This was partly echoed by Kumar et al., who found no difference in TAS levels between patients with *P. falciparum* and *P. vivax* malaria [30]. On the other hand, two studies [16, 34] found no significant difference in TAS levels between various patient groups and controls. Lastly, three studies on pregnant women showed diverse results [31, 35, 36]. Tiyong Ifoue et al*.* observed fluctuating TAS levels during different trimesters in pregnant women with malaria [35]. Mahamat et al*.* found a significant increase in TAS levels in patients with malaria compared to non-malaria cases [31]. Clinton et al. showed no difference in TAS levels between patients with malaria and non-malaria cases [36].

### Quantitative synthesis

**Fig. 2:**
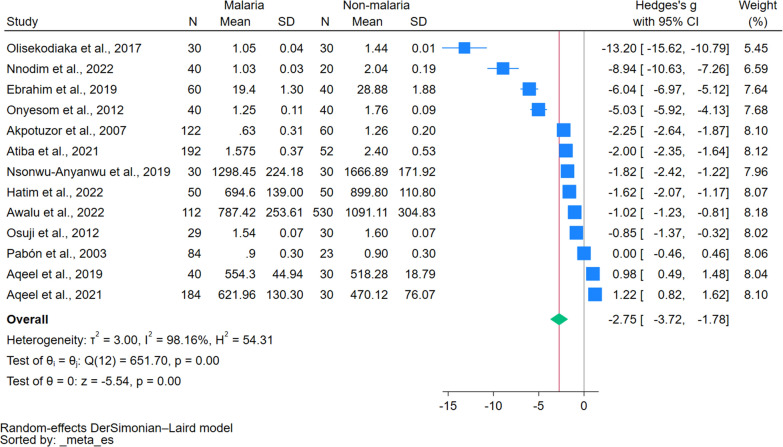
The forest plot shows the difference in TAS levels between malaria and non-malaria patients. Explanations for symbols: blue square, effect estimate of each study; green diamond, pooled effect estimate. *CI* confidence interval, *N* number of participants, *SD* standard deviation

Quantitative synthesis of TAS levels between malaria and non-malaria cases was conducted using meta-analysis. Of the total studies identified, 13 met the inclusion criteria for quantitative synthesis due to their sufficient data quality and relevance. The remaining 7 studies were excluded from the quantitative synthesis due to insufficient or inconsistent data. The meta-analysis showed a significant decrease in TAS levels in patients with malaria compared to non-malaria cases (*P* < 0.01, Hedges’ g: − 2.75, 95% CI − 3.72 to (− 1.78)), *I*^*2*^: 98.16%, 13 studies, Fig. 2). The results of a meta-regression analysis comparing TAS in malaria and non-malaria cases are shown in Table S4. Several covariates were examined for their effect on TAS levels, and the results showed that publication years and study design had a non-significant *P* value (*P* > 0.05), indicating a non-significant association with TAS levels. However, there were significant associations between continent (*P* = 0.018), age group (*P* = 0.004), *Plasmodium* species (*P* = 0.002), methods for measuring TAS (*P* = 0.037), and TAS levels, indicating a small proportion of the variance was explained by this covariate.

Table 2 presents a subgroup analysis comparing TAS levels between malaria and non-malaria cases, stratified by several subgroup categories. Under the publication year subgroup, studies from 2020–2023 and 2010–2019 exhibited significantly lower TAS levels in malaria cases (*P* < 0.01); however, studies from 2000–2009 showed no significant difference (*P* = 0.32). In the study design subgroup, case–control and cross-sectional studies presented significantly lower TAS levels in malaria cases (*P* < 0.01 and *P* = 0.01, respectively). The continent subgroup showed that TAS levels were significantly lower in Africa (*P* < 0.01), but not in Asia (*P* = 0.27). The country subgroup analysis revealed a significant difference in TAS levels for Nigeria (*P* < 0.01) but not for India (*P* = 0.83). In the age group subgroup, significantly lower TAS levels were found in adults (*P* = 0.01) but not in children (*P* = 0.16), all age groups (*P* = 0.05), or unspecified age groups (*P* = 0.43). In the *Plasmodium* species subgroup, *P. falciparum* showed significantly lower TAS levels (*P* < 0.01), while the *P. falciparum* and *P. vivax* subgroup did not (*P* = 0.32). The clinical symptom subgroup indicated significantly lower TAS levels in both symptomatic (*P* = 0.01) and unspecified (*P* < 0.01) categories. The diagnostic method for the malaria subgroup revealed that TAS levels were significantly lower in the microscopy method (*P* < 0.01) but not in other categories. Lastly, the subgroup analysis revealed that TAS levels were significantly lower when measured using TBARS (*P* = 0.03) and Trolox equivalent antioxidant capacity (TEAC) (*P* = 0.01).

**Table 2:** Subgroup analyses of TAS levels between malaria and non-malaria cases

Subgroup analyses	*P* value	Hedges’ g (95% CI)	I^2^ (%)	Number of studies
**Publication years**				
2020–2023	< 0.01	− 2.16 (− 3.52 to 0.79)	98.34	5
2010–2019	< 0.01	− 4.11 (− 6.54 to (− 1.68))	98.56	6
2000–2009	0.32	− 1.13 (− 3.33 to (− 1.08))	98.16	2
**Study design**				
Case–control studies	< 0.01	− 2.91 (− 4.42 to (− 1.40))	98.47	9
Cross-sectional studies	0.01	− 3.42 (− 5.98 to (− 0.86))	98.38	3
Cohort study	Not applicable	− 1.02 (− 12.23 to (− 0.81))	Not applicable	1
**Continent**				
Africa	< 0.01	− 3.76 (− 4.84 to (− 2.67))	97.42	8
Asia	0.27	− 1.33 (− 3.72 to 1.06)	98.85	4
South America	Not applicable	0.00 (− 0.46 to 0.46)	Not applicable	1
**Country**				
Nigeria	< 0.01	− 3.76 (− 4.84 to (− 2.67))	97.42	8
India	0.83	− 0.19 (− 1.61 to 2.00)	97.94	3
Ethiopia	Not applicable	− 6.04 (− 6.97 to (− 5.12))	Not applicable	1
Columbia	Not applicable	0.00 (− 0.46 to 0.46)	Not applicable	1
**Age group**				
Adults	0.01	− 1.26 (− 2.25 to (− 0.26))	97.31	7
Children	0.16	− 7.66 (− 18.39 to (− 3.07))	98.70	2
All age groups	0.05	− 4.00 (− 7.96 to (− 0.03))	98.44	2
Not specified age	0.43	− 3.94 (− 13.67 to 5.78)	99.18	2
***Plasmodium*** ***species***				
* P. falciparum*	< 0.01	− 4.62 (− 6.01 to (− 3.24))	97.41	8
* P. falciparum, P. vivax*	0.32	0.49 (− 0.48 to 1.45)	87.77	2
* P. falciparum, P. vivax,* mixed infections	Not applicable	1.62 (− 2.07 to 1.17)	Not applicable	1
* P. vivax*	Not applicable	1.22 (− 0.82 to 1.62)	Not applicable	1
Not specified	Not applicable	− 1.02 (− 1.23 to (− 0.81))	Not applicable	1
**Clinical symptoms**				
Symptomatic	0.01	− 1.38 (− 2.48 to (− 0.27))	97.73	7
Not specified	< 0.01	− 4.16 (− 5.54 to (− 2.79))	96.81	6
**Diagnostic method for malaria**				
Microscopy	< 0.01	− 3.57 (− 4.80 to (− 2.35))	97.48	8
Microscopy/RDT	0.46	0.46 (− 0.77 to 1.68)	95.05	3
Not specified	0.21	− 4.94 (− 12.70 to 2.82)	98.80	2
**Methods for measuring TAS levels**				
Ferric reducing antioxidant power (FRAP)	0.42	− 1.35 (− 4.63 to 1.94)	99.39	4
Thiobarbituric acid reactive substances (TBARS)	0.03	− 6.16 (− 11.85 to (− 0.46))	99.60	4
Trolox equivalent antioxidant capacity (TEAC)	0.01	− 3.61 (− 6.33 to (− 0.89))	96.81	2
2,2-azinobis 3-ethylbenzothiazoline-6-sulfonate	0.33	− 0.41 (− 1.24 to 0.42)	82.29	2
Commercial kits	Not applicable	− 2.00 (− 2.35 to (− 1.64))	Not applicable	1

*CI* confidence interval, *RDT* rapid diagnostic test

### Sensitivity analysis

**Fig. 3:**
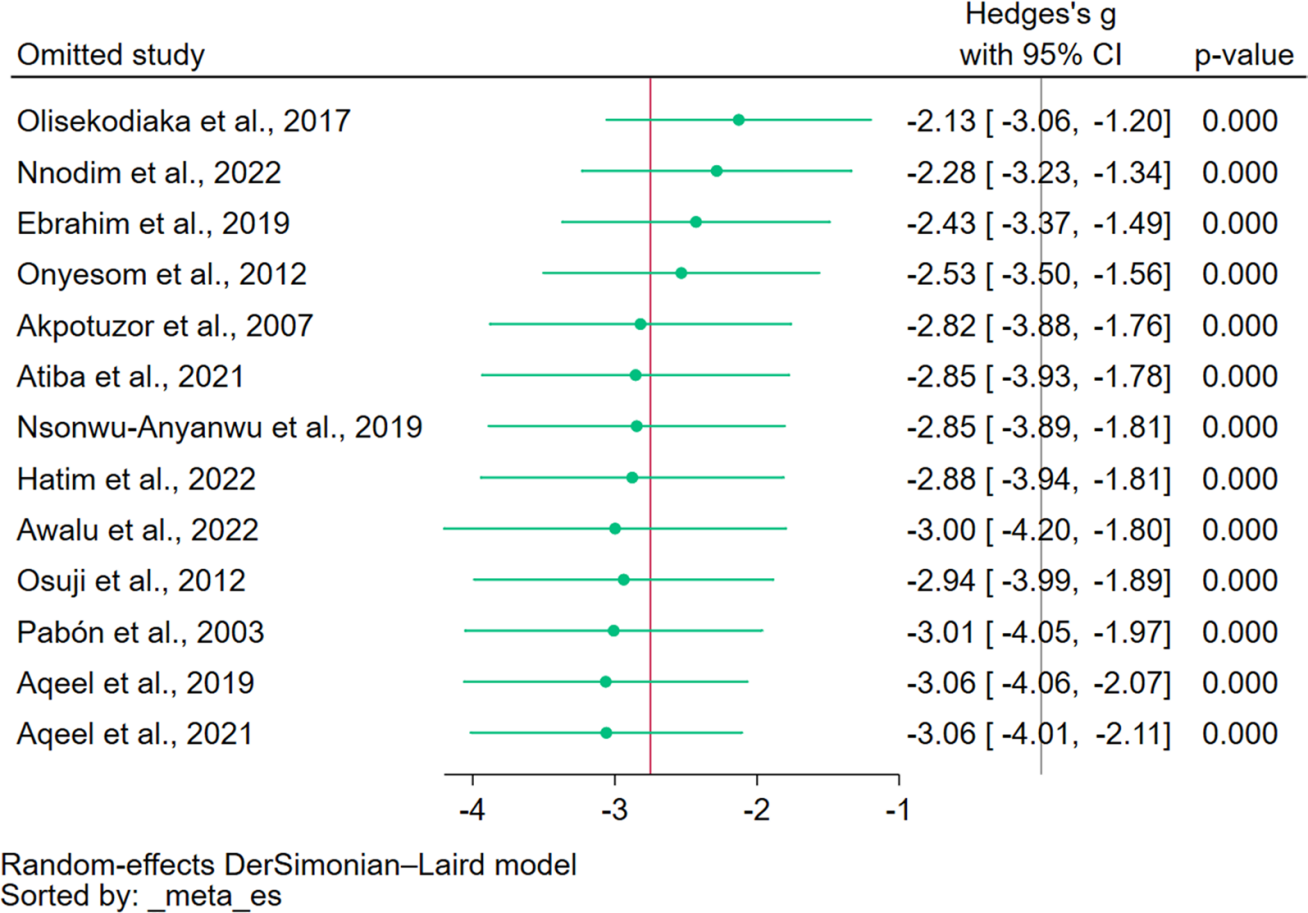
The leave-one-out analysis demonstrated the difference in TAS levels between malaria and non-malaria patients in each re-run analysis. Explanations for symbols: green dot, pooled effect estimate in each re-run analysis. *CI* confidence interval, *Mean Diff.* mean difference, *N* number of participants, *SD* standard deviation

The change in the statistical model for pooling the data was conducted. Using the fixed effect model showed a significant decrease in TAS levels in patients with malaria compared to non-malaria cases (*P* < 0.01, Hedges’ g: − 1.1765, 95% CI − 1.29 to (− 1.04)). The leave-one-out meta-analysis showed a significant decrease in TAS levels in patients with malaria compared to non-malaria cases in all re-runs of the analysis (*P* < 0.01, Fig. 3). The results of the sensitivity analyses demonstrated the robustness and stability of the meta-analysis results.

### Publication *bias*

**Fig. 4:**
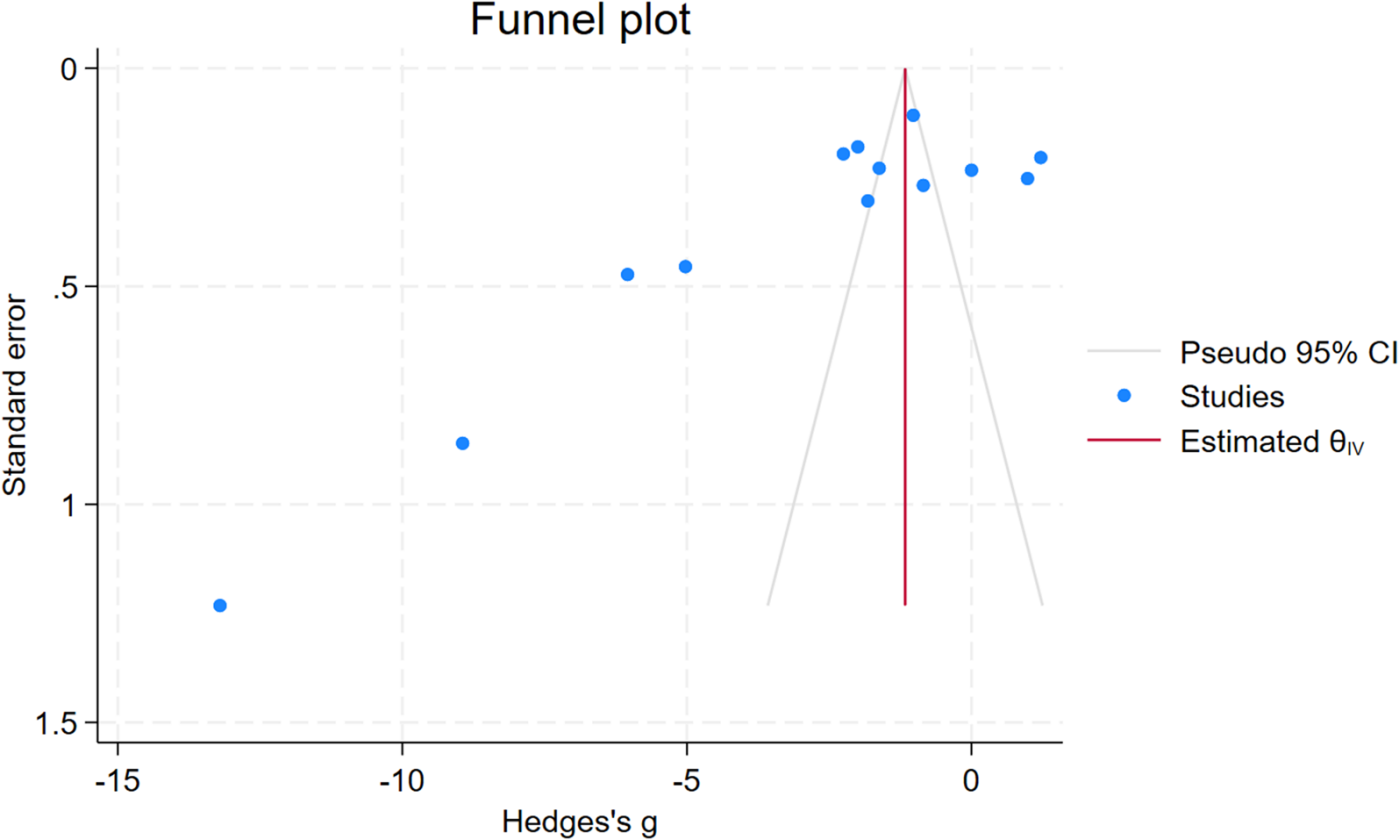
The funnel plot showed an asymmetrical distribution of the effect estimates (Hedges’ g, X-axis) and standard error (Y-axis). *CI* confidence interval

The funnel plot exhibited asymmetry (Fig. 4), suggesting a potential bias in the included studies. The regression-based Egger test for small-study effects yielded a significant result (*P* < 0.01), indicating that the observed funnel plot asymmetry might be attributed to the absence of small studies in the meta-analysis. Moreover, a nonparametric trim-and-fill analysis was conducted to assess publication bias further. This analysis indicated a decrease in TAS levels in patients with malaria compared to non-malaria cases (Hedges’ g: − 1.165, 95% CI − 1.288 to (− 1.043)). These results highlighted the potential impact of publication bias on the synthesized evidence and emphasized the importance of cautious interpretation when drawing conclusions from meta-analytical findings.

## Discussion

Peroxidation and antioxidant status evaluations can be significant markers for gauging a disease's risk, severity, or progression. Additionally, these evaluations can assess the efficacy of treatment interventions and therapies. The current meta-analysis presents substantial evidence from several studies denoting a marked decrease in TAS levels in patients with malaria compared to non-malaria cases. This trend was prominently observed across 14 studies [15, 17, 26, 27, 29, 32, 33, 35, 37–42], suggesting a potential link between malaria infection and lowered antioxidant levels, which might contribute to the severity of the disease. The depletion of TAS observed during malaria is a consequence of the disturbed balance between pro- and antioxidants, resulting from an excess accumulation of ROS and reactive nitrogen species (RNS), which commonly inflict damage on proteins and lipids in the serum of infected patients [28]. A decrease in TAS levels in malaria patients, as highlighted by the meta-analysis findings suggests an overwhelmed antioxidative response likely due to elevated oxidative stress induced by the infection.

Despite the high heterogeneity among studies, the meta-analysis revealed a significant decrease in TAS levels in patients with malaria compared to non-malaria cases. This meta-analysis also showed that certain factors, such as continent, age group, *Plasmodium* species, and method for measuring TAS, were significantly associated with TAS levels, suggesting these covariates could partially explain the variance in TAS levels among patients. Furthermore, the subgroup analysis reinforced the general findings and revealed intriguing patterns. Studies published more recently (2010–2023) and those with specific designs (case–control and cross-sectional) displayed lower TAS levels in malaria cases. This might be related to several factors, such as advancements in measurement techniques, an increased focus on oxidative stress in recent studies, or differences in study quality standards. Moreover, the association between decreased TAS levels and malaria was stronger in Africa than in Asia, which might be related to different malaria strains, healthcare contexts, nutrition, or genetic factors. The patient’s age also emerged as an essential factor, with adults showing a more pronounced decrease in TAS levels than children or non-specified age groups. This result might indicate differences in the antioxidant status of participants across age groups in the included studies. The diagnostic method for malaria seems to have an effect on TAS levels, with microscopy associated with lower TAS levels. Nevertheless, a few studies used other diagnostic methods for malaria; therefore, further research employing various diagnostic techniques, including molecular methods such as PCR and advanced imaging technologies, is necessary to validate and strengthen the findings of the subgroup analysis. Lastly, the method for measuring TAS seems to affect the observed TAS levels. The differences in TAS levels across these methods likely result from their distinct measurement principles, sensitivity to specific antioxidants, and interactions with sample components. FRAP’s broader measurement of antioxidant capacity [43] might partially explain why TAS levels did not show a significant decrease in malaria compared to the more specific methods like TBARS or TEAC.

Certain studies have elucidated a negative correlation between TAS levels and parasite density [17, 33, 40], suggesting that a high parasitic load may play a role in depleting the host's antioxidant status. It was observed that patients with high parasitaemia displayed a more marked decrease in TAS and an increase in total oxidative stress (TOS) compared to those with low to moderate parasitaemia [37]. This implies that the antioxidant resources were substantially consumed in the process of neutralizing the ROS produced during the course of the *Plasmodium* infection. After patients underwent treatment and parasites were cleared by anti-malarial drugs, TAS levels were higher. This increase, combined with lower parasite density, total plasma peroxide (TPP), and oxidative stress index (OSI), indicates a reduction in ROS generation by *Plasmodium* infections. Moreover, the negative association between TAS and parasite density suggests parasites damage red blood cells and alter antioxidant levels. This supposition is supported by the positive correlation found between TAS and hemoglobin levels in severe malaria cases [28]. A particularly interesting finding from the work of Aqeel et al*.* was the significant drop in TAS levels in severe malaria cases compared to non-severe ones [28], hinting that TAS could potentially serve as a predictor of malaria severity.

Understanding the functioning of endogenous and exogenous antioxidants within the blood of malaria patients is pivotal for interpreting TAS levels. Endogenous antioxidants, such as superoxide dismutase (SOD), catalase, and glutathione peroxidase [47], are produced within the body and form the primary defense against oxidative stress by neutralizing ROS [43]. Exogenous antioxidants derived from dietary sources (e.g., vitamins C and E and polyphenols) supplement these endogenous systems [44]. The dynamic interplay between these antioxidant defenses maintains oxidative balance under healthy conditions. However, during *Plasmodium* infection, the increased production of ROS can overwhelm these defenses, leading to oxidative stress and a measurable decrease in TAS levels. TAS reflects the cumulative antioxidant capacity of all endogenous and exogenous antioxidants in the blood, offering a comprehensive overview of the body's oxidative defense status. These individual antioxidants and enzymes contribute to the overall TAS measurement, providing insight into the oxidative stress status of malaria patients. Studies have shown that the primary defenses against oxidative stress, such as SOD [45], catalase [46], and glutathione peroxidase [47], are decreased in patients with malaria, indicating a compromised endogenous antioxidant system. Similarly, measurements of exogenous antioxidants in the blood have revealed reductions in vitamin C [48], vitamin E [49], and beta-carotene [50], further contributing to the decreased TAS levels observed in malaria patients. This integrated perspective on TAS levels, which encompasses both endogenous and exogenous antioxidants, highlights the complex nature of oxidative stress in malaria. It highlights the importance of understanding how these individual antioxidant parameters collectively influence TAS. Further investigations into the mechanisms underlying the observed changes in TAS levels are essential. Such studies should aim to elucidate the specific contributions of various antioxidants to TAS and explore targeted interventions to modulate oxidative stress, ultimately improving patient outcomes in malaria.

Several limitations were encountered in this study. First, the heterogeneity among the included studies was high, necessitating a cautious interpretation of the results. Second, a significant association between TAS levels and various factors, such as *Plasmodium* species, age, geographical location, and method for measuring TAS was found, which limited the power of the synthesis of findings. Third, most studies focused on adults, leaving a gap in understanding TAS levels in children and adolescents with malaria. Lastly, there was a notable geographic distribution bias in the included studies, with a significant concentration of research conducted in Nigeria. This presents a limitation regarding the generalizability of the findings across different malaria-endemic regions. This geographic imbalance highlights a crucial gap in literature. It highlights the need for more comprehensive research efforts that include underrepresented regions to fully understand the relationship between TAS levels and malaria across diverse environmental, genetic, and socio-economic contexts. Future studies should aim to standardize methodologies, control potential confounders, and include underrepresented and vulnerable populations to provide a more comprehensive understanding of the relationship between malaria and TAS levels.

## Conclusion

This systematic review and meta-analysis highlighted a significant decrease in TAS levels among malaria patients compared to non-malaria cases, suggesting that TAS levels could be a helpful marker in malaria diagnosis. Additionally, the findings indicated that factors such as *Plasmodium* species, age, geographical location, and method for measuring TAS significantly influenced TAS levels. Regarding the variety of study designs among the included studies, future prospective cohort studies should be conducted to produce more reliable and comprehensive insights into the role of oxidative stress in malaria, particularly the specific mechanisms by which *Plasmodium* infection influences TAS levels and explore the potential of TAS as a predictor of malaria severity.

### Supplementary Information

Supplementary file 1.

Supplementary file 2.

Supplementary file 3.

Supplementary file 4.

## Data Availability

All data relating to the present study are available in this manuscript and supplementary files.
