# Supplementary material for: Total antioxidant status levels in malaria: a systematic review and meta-analysis
Source: Malar J. 2024 Jun 26;23:198. doi: 10.1186/s12936-024-05003-z (PMC11210049; doi:10.1186/s12936-024-05003-z)
Supplement: Supplementary file 1 [file 12936_2024_5003_MOESM1_ESM.docx]

**Total antioxidant status levels in malaria: A systematic review and meta-analysis**

Kwuntida Uthaisar Kotepui^1^, Aongart Mahittikorn^2^, Wanida Mala^1^, Supakanya Lasom^3^, Frederick Ramirez Masangkay^4^, Hideyuki J Majima^5^, Manas Kotepui^1^*

^1^Medical Technology, Faculty of Science, Nakhon Phanom University, Nakhon Phanom 48000

^2^Department of Protozoology, Faculty of Tropical Medicine, Mahidol University, Bangkok, Thailand

^3^School of Allied Health Sciences, University of Phayao

^4^Department of Medical Technology, Faculty of Pharmacy, University of Santo Tomas, Manila, Philippines

^5^Medical Technology, School of Allied Health Sciences, Walailak University, Tha Sala, Nakhon Si Thammarat, Thailand

*Corresponding author

Kwuntida Uthaisar Kotepui: kwunta@hotmail.com

Aongart Mahittikorn: [aongart.mah@mahidol.ac.th](mailto:aongart.mah@mahidol.ac.th)

Wanida Mala: wanida.maa@gmail.com

Supakanya Lasom: supakanya.la@up.ac.th

Hideyuki J Majima: [k0941761@kadai.jp](mailto:k0941761@kadai.jp)

Frederick Ramirez Masangkay: frmasangkay@ust.edu.ph

Manas Kotepui: [manaskote@gmail.com](mailto:manaskote@gmail.com), Tel.: +66954392469

**General keywords**

(“total antioxidant status” OR TAS OR “oxidative stress index” OR OSI OR “total antioxidant capacity” OR TAC OR “redox status” OR “total antioxidant” OR “total oxidant” OR “total oxidant status” OR TOS OR “total antioxidant defense” OR “total antioxidant power” OR “Ferric Reducing Ability of Plasma” OR FRAP OR “overall antioxidant status” OR ”overall antioxidant capacity” OR ”overall antioxidant power” OR ”overall antioxidant defense”) AND (malaria OR plasmodium OR “Plasmodium Infection“ OR “Remittent Fever“ OR “Marsh Fever“ OR Paludism)

PubMed 25 July 2023

| No. | Key concept | Search terms | Results |
| --- | --- | --- | --- |
| 1. | Total antioxidant status | (“total antioxidant status”[Text Word] OR “total antioxidant status”[MeSH Terms] OR TAS[Text Word] OR “oxidative stress index”[Text Word] OR “oxidative stress index”[MeSH Terms] OR OSI[Text Word] OR “total antioxidant capacity”[Text Word] OR “total antioxidant capacity”[MeSH Terms] OR TAC[Text Word] OR “redox status”[Text Word] OR “redox status” [MeSH Terms] OR “total antioxidant”[Text Word] OR “total oxidant”[Text Word] OR “total oxidant status”[Text Word] OR TOS[Text Word] OR “total antioxidant defense”[Text Word] OR “total antioxidant power”[Text Word] OR “Ferric Reducing Ability of Plasma”[Text Word] OR FRAP[Text Word] OR “overall antioxidant status” [Text Word] OR ”overall antioxidant capacity” [Text Word] OR ”overall antioxidant power”[Text Word] OR ”overall antioxidant defense”[Text Word]) | 51,159 |
| 2. | Malaria | malaria[Text Word] OR plasmodium[Text Word] OR malaria[MeSH Terms] OR “Remittent Fever“[Text Word] OR “Marsh Fever“[Text Word] OR Paludism[Text Word] | 123,104 |
| 3. | 1 AND 2 | (“total antioxidant status”[Text Word] OR “total antioxidant status”[MeSH Terms] OR TAS[Text Word] OR “oxidative stress index”[Text Word] OR “oxidative stress index”[MeSH Terms] OR OSI[Text Word] OR “total antioxidant capacity”[Text Word] OR “total antioxidant capacity”[MeSH Terms] OR TAC[Text Word] OR “redox status”[Text Word] OR “redox status” [MeSH Terms] OR “total antioxidant”[Text Word] OR “total oxidant”[Text Word] OR “total oxidant status”[Text Word] OR TOS[Text Word] OR “total antioxidant defense”[Text Word] OR “total antioxidant power”[Text Word] OR “Ferric Reducing Ability of Plasma”[Text Word] OR FRAP[Text Word] OR “overall antioxidant status” [Text Word] OR ”overall antioxidant capacity” [Text Word] OR ”overall antioxidant power”[Text Word] OR ”overall antioxidant defense”[Text Word]) AND (malaria[Text Word] OR plasmodium[Text Word] OR malaria[MeSH Terms] OR “Remittent Fever“[Text Word] OR “Marsh Fever“[Text Word] OR Paludism[Text Word]) | 108 |

Embase 25 July 2023

| No. | Key concept | Search terms | Results |
| --- | --- | --- | --- |
| 1. | Total antioxidant status | (“total antioxidant status”:ti,ab,kw,de OR “total antioxidant status”/exp OR TAS:ti,ab,kw,de OR “oxidative stress index”:ti,ab,kw,de OR “oxidative stress index”/exp OR OSI:ti,ab,kw,de OR “total antioxidant capacity”:ti,ab,kw,de OR “total antioxidant capacity”/exp OR TAC:ti,ab,kw,de OR “redox status”:ti,ab,kw,de OR “redox status”/exp OR “total antioxidant”:ti,ab,kw,de OR “total oxidant”:ti,ab,kw,de OR “total oxidant status”:ti,ab,kw,de OR TOS:ti,ab,kw,de OR “total antioxidant defense”:ti,ab,kw,de OR “total antioxidant power”:ti,ab,kw,de OR “Ferric Reducing Ability of Plasma”:ti,ab,kw,de OR FRAP:ti,ab,kw,de OR “overall antioxidant status”:ti,ab,kw,de OR ”overall antioxidant capacity”:ti,ab,kw,de OR ”overall antioxidant power”:ti,ab,kw,de OR ”overall antioxidant defense”:ti,ab,kw,de) | 72,405 |
| 2. | Malaria | malaria:ti,ab,kw,de OR plasmodium:ti,ab,kw,de OR ‘Remittent Fever’:ti,ab,kw,de OR ‘Marsh Fever’:ti,ab,kw,de OR Paludism:ti,ab,kw,de OR malaria/exp | 156,896 |
| 3. | 1 AND 2 | (“total antioxidant status”:ti,ab,kw,de OR “total antioxidant status”/exp OR TAS:ti,ab,kw,de OR “oxidative stress index”:ti,ab,kw,de OR “oxidative stress index”/exp OR OSI:ti,ab,kw,de OR “total antioxidant capacity”:ti,ab,kw,de OR “total antioxidant capacity”/exp OR TAC:ti,ab,kw,de OR “redox status”:ti,ab,kw,de OR “redox status”/exp OR “total antioxidant”:ti,ab,kw,de OR “total oxidant”:ti,ab,kw,de OR “total oxidant status”:ti,ab,kw,de OR TOS:ti,ab,kw,de) AND (malaria:ti,ab,kw,de OR plasmodium:ti,ab,kw,de OR ‘Remittent Fever’:ti,ab,kw,de OR ‘Marsh Fever’:ti,ab,kw,de OR Paludism:ti,ab,kw,de OR malaria/exp) | 158 |

Scopus 25 July 2023

| No. | Key concept | Search terms | Results |
| --- | --- | --- | --- |
| 1. | Total antioxidant status | TITLE-ABS-KEY (“total antioxidant status” OR TAS OR “oxidative stress index” OR OSI OR “total antioxidant capacity” OR TAC OR “redox status” OR “total antioxidant” OR “total oxidant” OR “total oxidant status” OR TOS OR “total antioxidant defense” OR “total antioxidant power” OR “Ferric Reducing Ability of Plasma” OR FRAP OR “overall antioxidant status” OR ”overall antioxidant capacity” OR ”overall antioxidant power” OR ”overall antioxidant defense”) | 99,160 |
| 2. | Malaria | TITLE-ABS-KEY ( ( malaria OR plasmodium OR "plasmodium infection" OR "remittent fever" OR "marsh fever" OR paludism ) ) | 157,694 |
| 3. | 1 AND 2 | ( TITLE-ABS-KEY (“total antioxidant status” OR TAS OR “oxidative stress index” OR OSI OR “total antioxidant capacity” OR TAC OR “redox status” OR “total antioxidant” OR “total oxidant” OR “total oxidant status” OR TOS OR “total antioxidant defense” OR “total antioxidant power” OR “Ferric Reducing Ability of Plasma” OR FRAP OR “overall antioxidant status” OR ”overall antioxidant capacity” OR ”overall antioxidant power” OR ”overall antioxidant defense”) AND (malaria OR plasmodium OR “Plasmodium Infection“ OR “Remittent Fever“ OR “Marsh Fever“ OR Paludism) | 166 |

MEDLINE 25 July 2023

| No. | Key concept | Search terms | Results |
| --- | --- | --- | --- |
| 1. | Total antioxidant status AND Malaria | (“total antioxidant status” OR TAS OR “oxidative stress index” OR OSI OR “total antioxidant capacity” OR TAC OR “redox status” OR “total antioxidant” OR “total oxidant” OR “total oxidant status” OR TOS OR “total antioxidant defense” OR “total antioxidant power” OR “Ferric Reducing Ability of Plasma” OR FRAP OR “overall antioxidant status” OR ”overall antioxidant capacity” OR ”overall antioxidant power” OR ”overall antioxidant defense”) AND (malaria OR plasmodium OR “Plasmodium Infection“ OR “Remittent Fever“ OR “Marsh Fever“ OR Paludism) | 126 |

Ovid 25 July 2023

| No. | Key concept | Search terms | Results |
| --- | --- | --- | --- |
| 1. | Total antioxidant status AND Malaria | (“total antioxidant status” OR TAS OR “oxidative stress index” OR OSI OR “total antioxidant capacity” OR TAC OR “redox status” OR “total antioxidant” OR “total oxidant” OR “total oxidant status” OR TOS OR “total antioxidant defense” OR “total antioxidant power” OR “Ferric Reducing Ability of Plasma” OR FRAP) AND (malaria OR plasmodium OR “Plasmodium Infection“ OR “Remittent Fever“ OR “Marsh Fever“ OR Paludism)  Limit to (ovid full text available and articles with abstracts and original articles) | 146 |

ProQuest 25 July 2023

| No. | Key concept | Search terms | Results |
| --- | --- | --- | --- |
| 1. | Total antioxidant status AND Malaria | (“total antioxidant status” OR TAS OR “oxidative stress index” OR OSI OR “total antioxidant capacity” OR TAC OR “redox status” OR “total antioxidant” OR “total oxidant” OR “total oxidant status” OR TOS OR “total antioxidant defense” OR “total antioxidant power” OR “Ferric Reducing Ability of Plasma” OR FRAP OR “overall antioxidant status” OR ”overall antioxidant capacity” OR ”overall antioxidant power” OR ”overall antioxidant defense”) AND (malaria OR plasmodium OR “Plasmodium Infection“ OR “Remittent Fever“ OR “Marsh Fever“ OR Paludism) | 1092 |
