# Supplementary material for: Total antioxidant status levels in malaria: a systematic review and meta-analysis
Source: Malar J. 2024 Jun 26;23:198. doi: 10.1186/s12936-024-05003-z (PMC11210049; doi:10.1186/s12936-024-05003-z)
Supplement: Supplementary file 4 [file 12936_2024_5003_MOESM4_ESM.docx]

**Total antioxidant status levels in malaria: A systematic review and meta-analysis**

Kwuntida Uthaisar Kotepui^1^, Aongart Mahittikorn^2^, Wanida Mala^1^, Supakanya Lasom^3^, Frederick Ramirez Masangkay^4^, Hideyuki J Majima^5^, Manas Kotepui^1^*

^1^Medical Technology, Faculty of Science, Nakhon Phanom University, Nakhon Phanom 48000

^2^Department of Protozoology, Faculty of Tropical Medicine, Mahidol University, Bangkok, Thailand

^3^School of Allied Health Sciences, University of Phayao

^4^Department of Medical Technology, Faculty of Pharmacy, University of Santo Tomas, Manila, Philippines

^5^Medical Technology, School of Allied Health Sciences, Walailak University, Tha Sala, Nakhon Si Thammarat, Thailand

*Corresponding author

Kwuntida Uthaisar Kotepui: kwunta@hotmail.com

Aongart Mahittikorn: [aongart.mah@mahidol.ac.th](mailto:aongart.mah@mahidol.ac.th)

Wanida Mala: wanida.maa@gmail.com

Supakanya Lasom: supakanya.la@up.ac.th

Hideyuki J Majima: [k0941761@kadai.jp](mailto:k0941761@kadai.jp)

Frederick Ramirez Masangkay: frmasangkay@ust.edu.ph

Manas Kotepui: [manaskote@gmail.com](mailto:manaskote@gmail.com), Tel.: +66954392469

**Table S4. Meta-regression results**

| **Meta-analysis of TAS** | **Covariates** | ***P* value** | **tau2** | **I^2^ (%)** | **R-squared (%)** |  |
| --- | --- | --- | --- | --- | --- | --- |
| **Malaria vs. non-malaria cases** | Publication years | 0.175 | 3.863 | 98.44 | 0 | 13 |
|  | Study design | 0.653 | 5.061 | 98.45 | 0 | 13 |
|  | Country | N/A | N/A | N/A | N/A | 13 |
|  | Continent | 0.018 | 3.17 | 98.12 | 0 | 13 |
|  | Age group | 0.004 | 3.477 | 98.15 | 0 | 13 |
|  | *Plasmodium* species | <0.001 | 3.062 | 97.13 | 0 | 13 |
|  | Clinical symptoms | N/A | N/A | N/A | N/A | 13 |
|  | Diagnostic method for malaria | 0.001 | 3.005 | 97.51 | 00.189 | 13 |
|  | Methods for measuring TAS | 0.037 | 5.201 | 98.33 | 0 | 13 |

N/A, not assessed because of collinearity; TAS, total antioxidant status
